# Supplementary material for: Liquid metal-embraced photoactive films for artificial photosynthesis
Source: Nat Commun. 2024 Feb 23;15:1672. doi: 10.1038/s41467-024-46073-6 (PMC10891066; doi:10.1038/s41467-024-46073-6)
Supplement: Supplementary file 1 — Supplementary information [file 41467_2024_46073_MOESM1_ESM.pdf]

Supplementary Materials

Liquid metal-embraced photoactive films for artificial photosynthesis

Chao Zhen[1,10#], Xiangtao Chen[2#], Ruotian Chen[3], Fengtao Fan[3], Xiaoxiang Xu[4], Yuyang Kang[1], Jingdong Guo[1], Lianzhou Wang[5], Gao Qing (Max) Lu[6], Kazunari Domen[7,8], Hui-Ming Cheng[1,9] & Gang Liu[1,10]*

[1]Shenyang National Laboratory for Materials Science, Institute of Metal Research, Chinese Academy of Sciences, 72 Wenhua Road, Shenyang 110016, China.

[2]Key Laboratory for Anisotropy and Texture of Materials (Ministry of Education), Northeastern University, Shenyang, Liaoning, 110819, China.

[3]State Key Laboratory of Catalysis, Dalian National Laboratory for Clean Energy, iChEM, Dalian Institute of Chemical Physics, Chinese Academy of Sciences, Dalian, China.

[4]School of Chemical Science and Engineering, Tongji University, Shanghai, 200092, China.

[5]Nanomaterials Centre, School of Chemical Engineering and AIBN, The University of Queensland, St Lucia, Brisbane, QLD, 4072, Australia.

[6]University of Surrey, Guilford, GU2 7XH UK.

[7]Research Initiative for Supra-Materials, Shinshu University, Nagano, Japan.

[8]Office of University Professors, The University of Tokyo, Tokyo, Japan.

[9]Institute of Technology for Carbon Neutrality, Shenzhen Institute of Advanced Technology, Chinese Academy of Sciences, 1068 Xueyuan Blvd, Shenzhen 518055, China.

[10]School of Materials Science and Engineering, University of Science and Technology of China, 72 Wenhua Road, Shenyang 110016, China.

[#] These authors contributed equally; *Correspondence: gangliu@imr.ac.cn

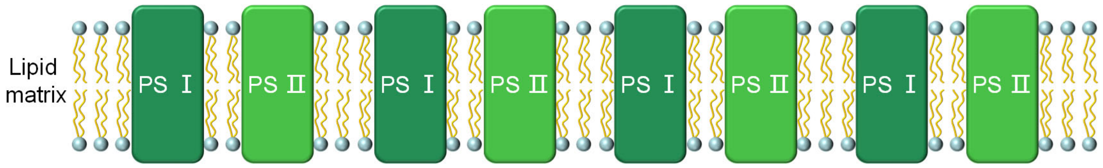

Fig. S1. Schematic of photosynthetic pigment-protein complexes (photosystem I/II) embedded in the lipid matrix of thylakoid membrane of chloroplasts in plant.

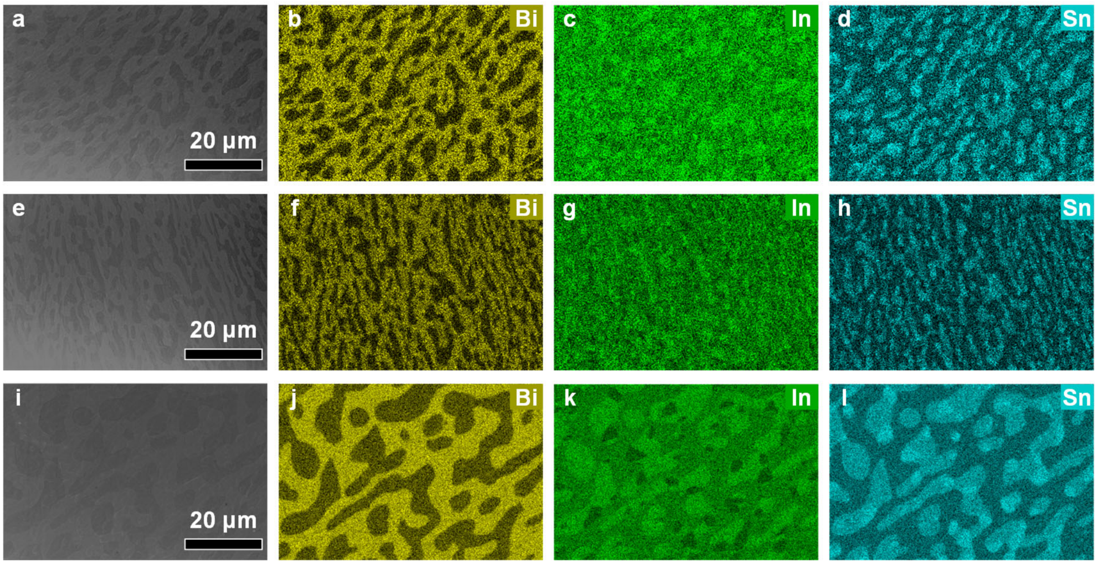

Fig. S2. The top-viewed SEM images and corresponding EDS mappings of Field's metal films coated on (a-d) Si, (e-h) FTO, and (i-l) PET substrates.

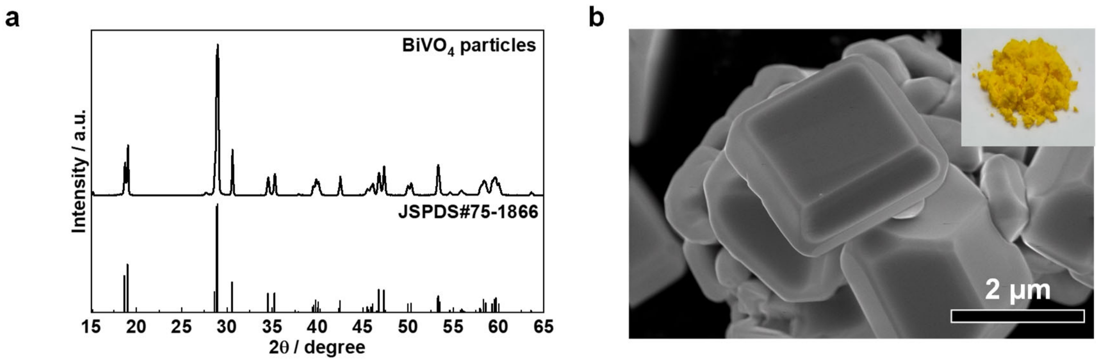

Fig. S3. (a) XRD patterns of synthesized $BiVO_4$ powder used in this study. (b) SEM image of synthesized $BiVO_4$ powder and the inset is its optical image.

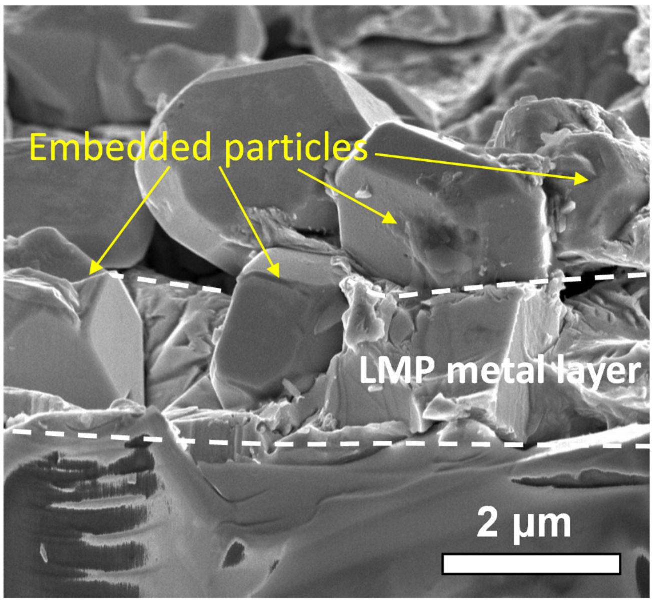

Fig. S4. The cross-sectional SEM image of the LMP metal embraced $BiVO_4$ photoelectrode.

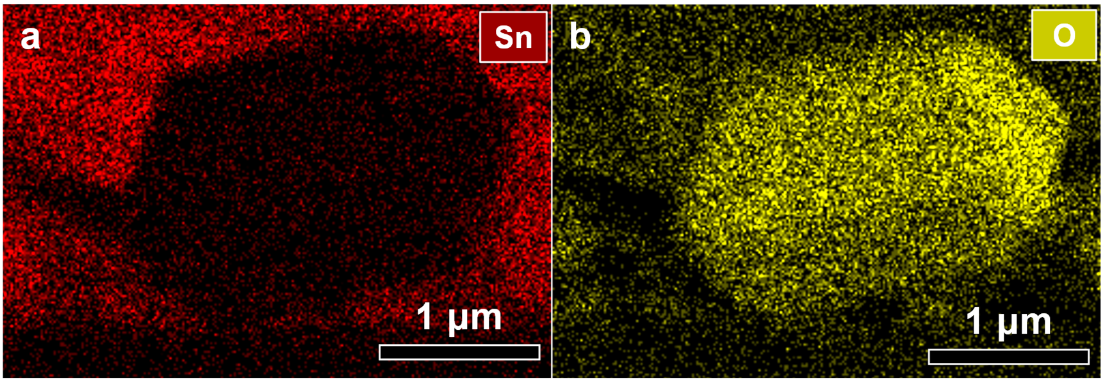

Fig. S5. The EDS mapping images of (a) Sn and (b) O recorded from the cross-sectional SEM image of the liquid metal-supported $BiVO_4$ photoelectrode.

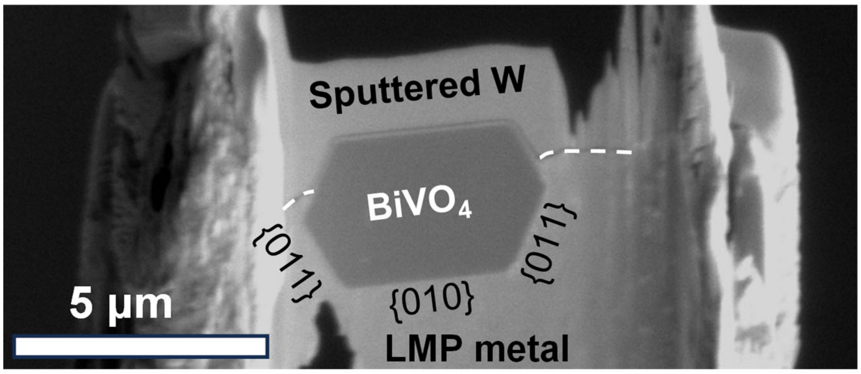

Fig. S6. The sectional view of FIB cutting of a representative particle of $BiVO_4$ with well-developed facets together with the LMP metal.

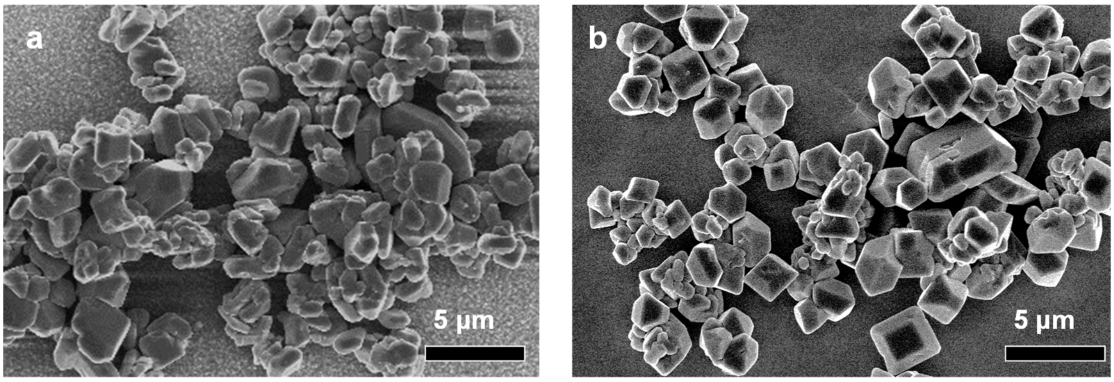

Fig. S7. The SEM images of the control $BiVO_4$ photoelectrodes assembled on the surface of (a) FTO substrate and (b) the LMP metal film.

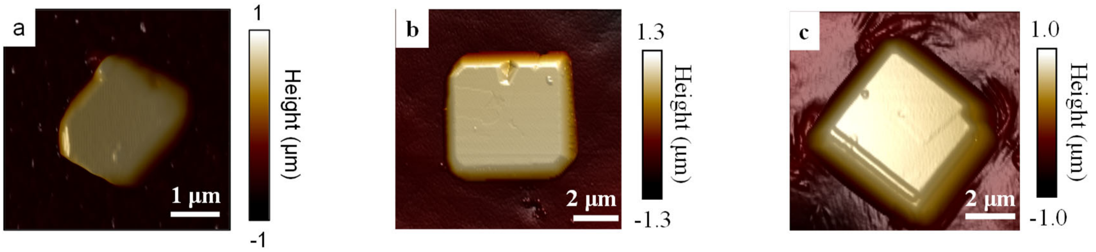

Fig. S8. The AFM images of typical $BiVO_4$ particles with well-developed facets assembled on (a) FTO, (b) on the surface of LMP metal film and (c) embedded in the LMP metal film, respectively.

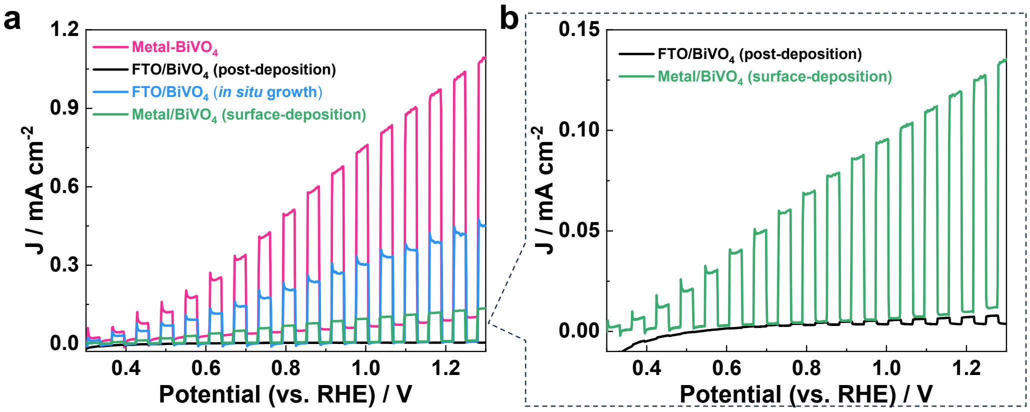

Fig. S9. (**a**), the polar J-V curves of the LMP metal supported $BiVO_4$ photoelectrode film (Metal/$BiVO_4$ (*surface-deposition*)), LMP metal embraced $BiVO_4$ photoelectrode film (Metal-$BiVO_4$) and the FTO-supported $BiVO_4$ photoelectrode using conventional particle deposition technique (FTO/$BiVO_4$ (*post-deposition*)) and FTO-supported $BiVO_4$ photoelectrode using hydrothermal growth method (FTO/$BiVO_4$ (*in situ growth*)), and (**b**) comparison of the polar J-V curves of the control photoelectrodes: FTO supported $BiVO_4$ film using conventional deposition technique (FTO/$BiVO_4$ (*post-deposition*)) and the LMP metal supported $BiVO_4$ film (Metal/$BiVO_4$ (*surface-deposition*)).

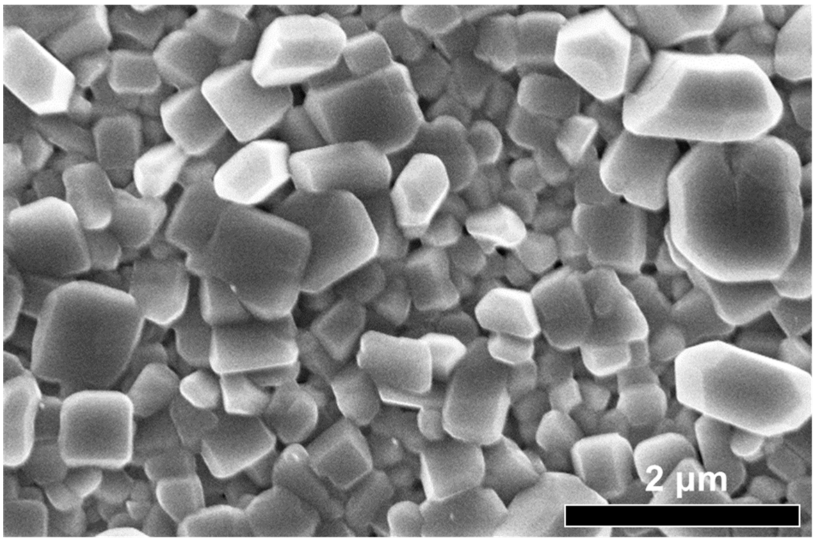

Fig. S10. The SEM image of the control $BiVO_4$ photoelectrode *in situ* grown on FTO.

Table S1 Comparison of the photoelectrochemical water splitting performance between the $BiVO_4$ particles-embedded photoelectrode and the typical $BiVO_4$ photoelectrodes *in situ* grown on FTO substrates with similar particle sizes (all $BiVO_4$ was undoped).

Morphology	Particle Size	Onset potential (V vs. RHE)	J_{SC} at 1.23 V_{RHE} ($mA\ cm^{-2}$)	Electrolyte	Ref.
Single crystal plate arrays	{010} basal: 1.5 μm<br>{010} normal: 650 nm	0.27	0.94	0.5 M Na_2SO_4 (pH 6.8)	[1]
Nanoplates	{010} basal: 550 nm<br>{010} normal: 300 nm	0.3	0.25	1 M potassium borate (pH 9.5)	[2]
Nanoplate arrays	{010} basal: 1.5 μm<br>{010} normal: 150 nm	0.7	1.41	0.2 M phosphate buffer (pH 7)	[3]
[010]- orientated microplates	{010} basal: 1.25 μm<br>{010} normal: 2.2 μm	0.5	0.61	1 M potassium borate (pH 9)	[4]
Microparticles	{010} basal: 2 μm<br>{010} normal: 1 μm	0.2	1.0	1 M potassium borate (pH 9)	This work

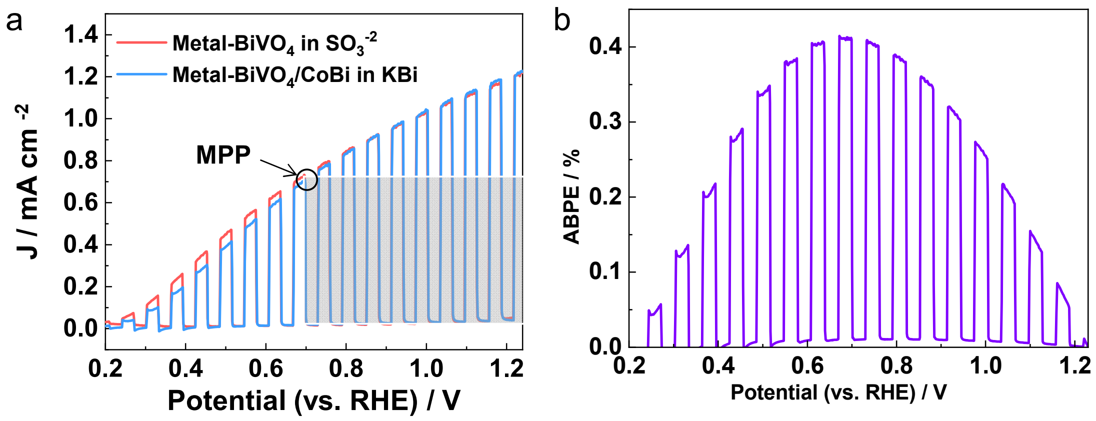

Fig. S11. (a) The polar *J-V* curves of the $BiVO_4$ particles-embedded photoelectrode film in aqueous electrolyte with the presence of hole sacrificial regent (SO_3^{2-}) and the

$BiVO_4$ particles-embedded photoelectrode film modified with the cobalt borate (CoBi) oxygen evolution catalyst in aqueous solution without hole sacrificial regent. (b) The applied bias photon-to-current conversion efficiency (ABPE) spectrum of the $BiVO_4$ particles-embedded photoelectrode after surface modification of CoBi.

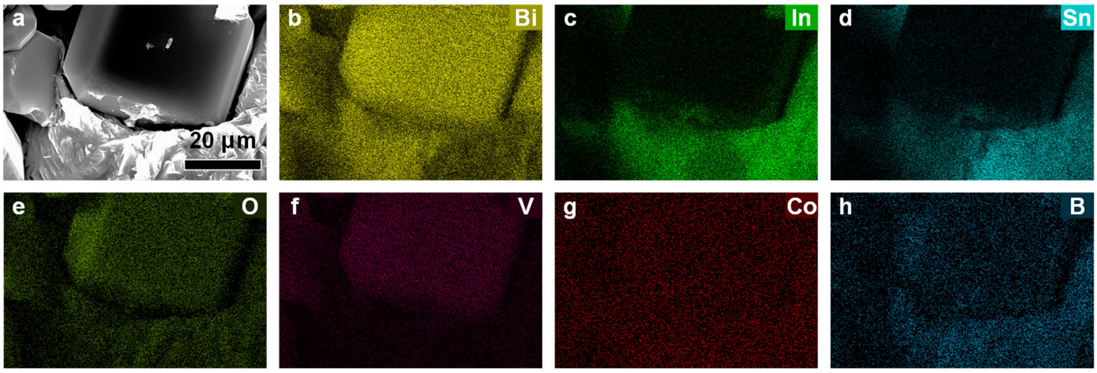

Fig. S12. (a) The top-viewed SEM images of the $BiVO_4$ particles-embedded photoelectrode with CoBi decoration and corresponding EDS mappings of (b) Bi, (c) In, (d) Sn, (e) O, (f) V, (g) Co and (h) B.

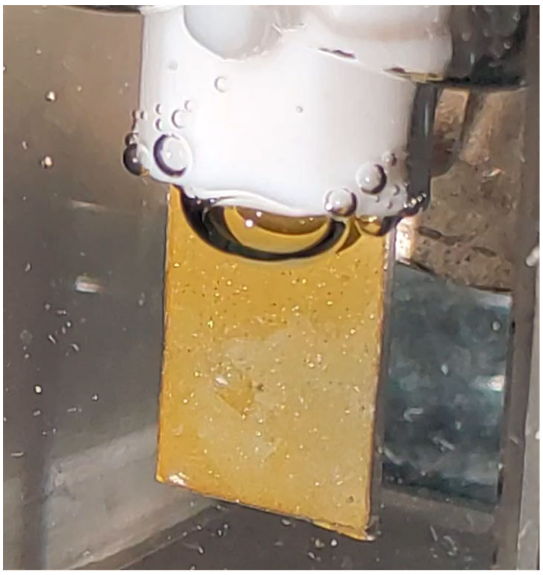

Fig. S13. The optical image of the $BiVO_4$ particles-embedded photoelectrode during the operation.

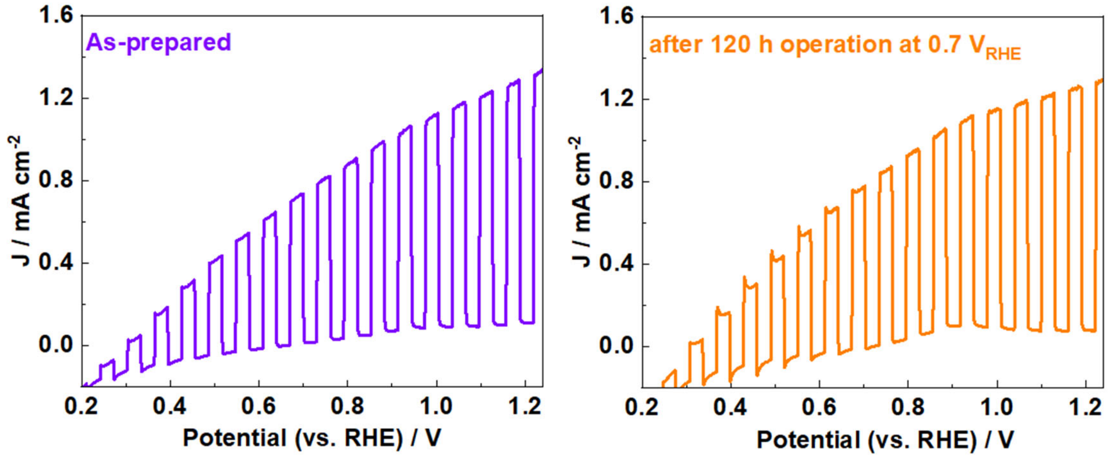

Fig. S14. The polar J-V curves of the $BiVO_4$ particles embedded photoelectrode before and after the stability test.

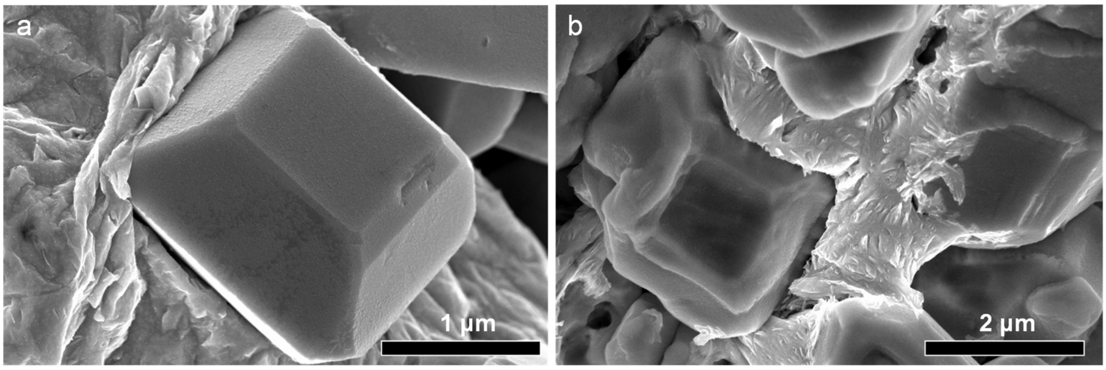

Figure S15. The SEM images of the CoBi cocatalyst decorated $BiVO_4$ particles-embedded photoelectrode (a) before and (b) after the stability test.

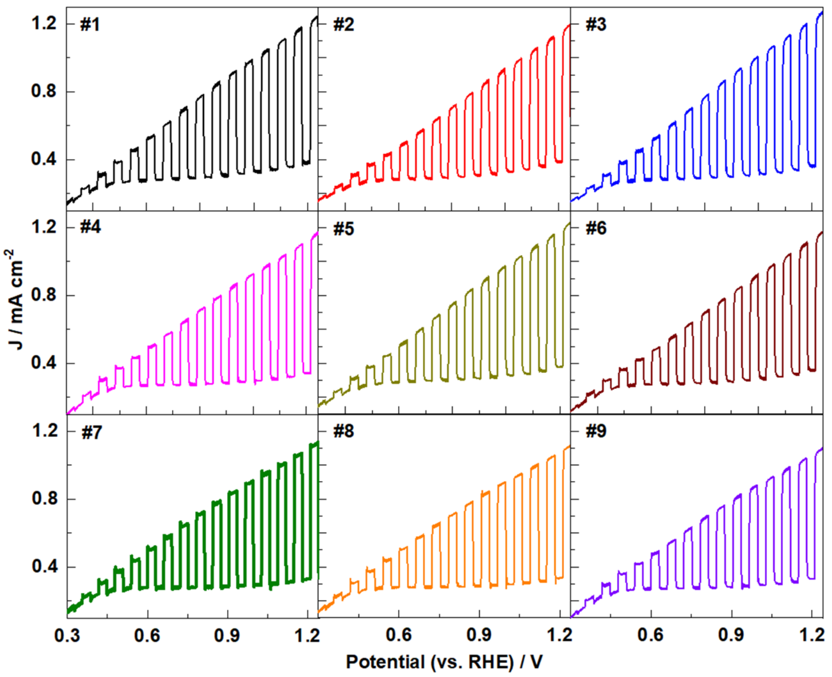

Fig. S16. The polar J-V curves recorded from the nine local regions of the $BiVO_4$ particles-embedded film of 10×10 cm^2 assembled on the quartz substrate, including its center (5#), sides (2#, 4#, 6#, 8#) and corners (1#, 3#, 7#, 9#).

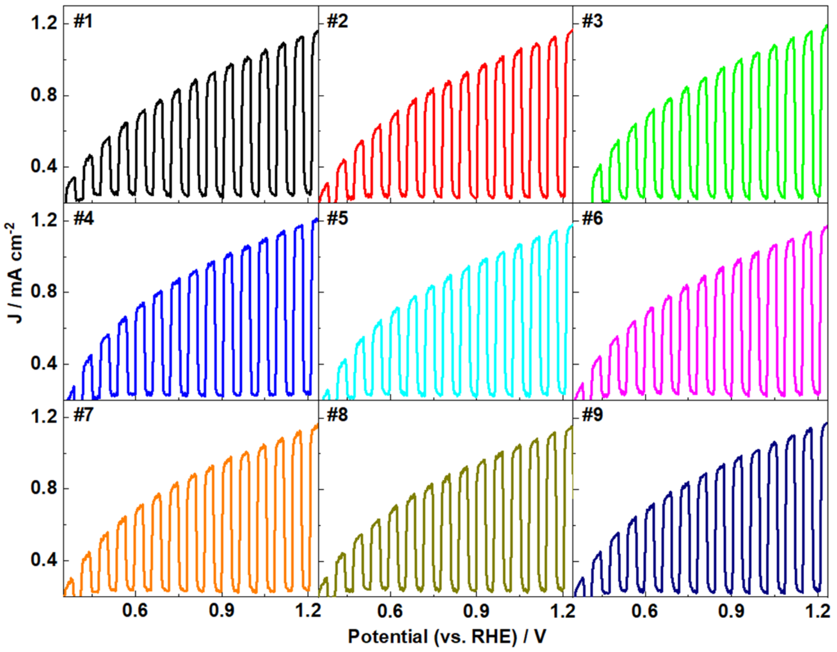

Fig. S17. The polar J-V curves recorded from the nine local regions of the $BiVO_4$ particles-embedded photoelectrode film of 10×10 cm^2 assembled on the quartz substrate after decorating CoBi, including its center (#5), sides (#2, 4, 6, 8) and corners (#1, 3, 7, 9).

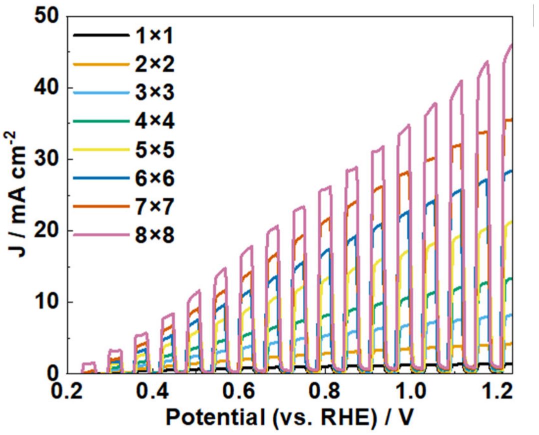

Fig. S18. The polar I-V curves recorded on the $BiVO_4$ particles-embedded photoelectrodes of 10×10 cm^2 after the CoBi decoration with different irradiation areas (1×1, 2×2, 3×3, 4×4, 5×5, 6×6, 7×7 and 8×8 cm^2) at its central part.

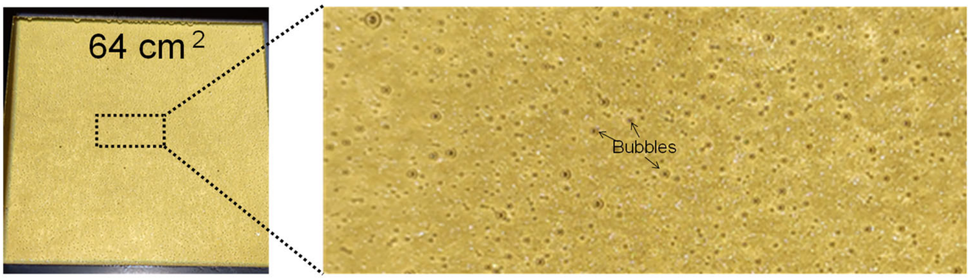

Fig. S19. The optical images of the irradiated area of the photoelectrode and its zoom-in image with bubble evolutions,

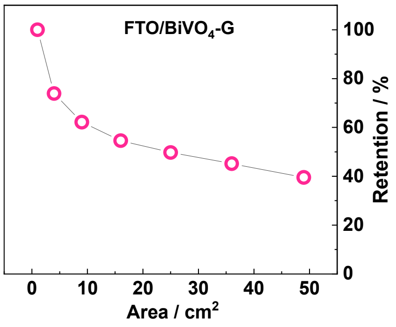

Fig. S20. The photocurrent density retentions of operation zones with different areas recorded on the control faceted $BiVO_4$ photoanode *in situ* grown on FTO (FTO/$BiVO_4$-G) at 1.23 V_{RHE}.

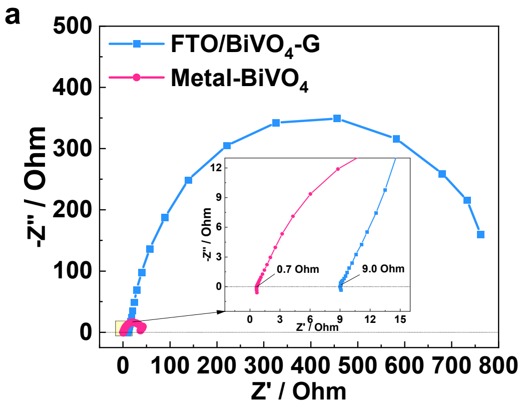

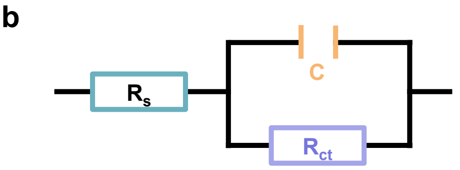

c

Samples	R_s (Ω)	R_{ct} (Ω)	C (mF)
FTO-$BiVO_4$-G	9.86	712.40	0.29
Metal-$BiVO_4$	0.76	35.54	1.05

Fig. S21. (a) The impedance spectra collected from Metal-$BiVO_4$ and FTO/$BiVO_4$-G photoanodes. (b) The equivalent circuit adopted for the simulation of photoelectrodes. (c) The simulation results of the impedance spectra collected from Metal-$BiVO_4$ and FTO/$BiVO_4$-G photoanodes.

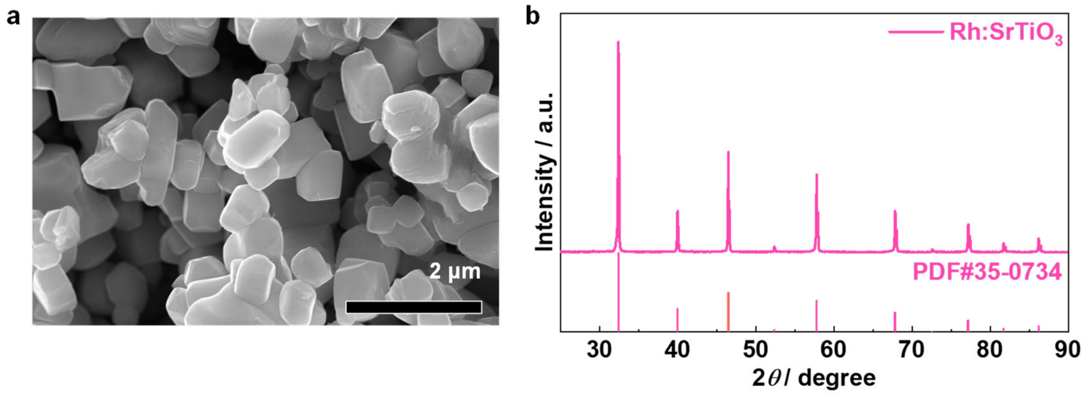

Fig. S22. (a) SEM image and (b) XRD patterns of synthesized Rh:$SrTiO_3$ powders.

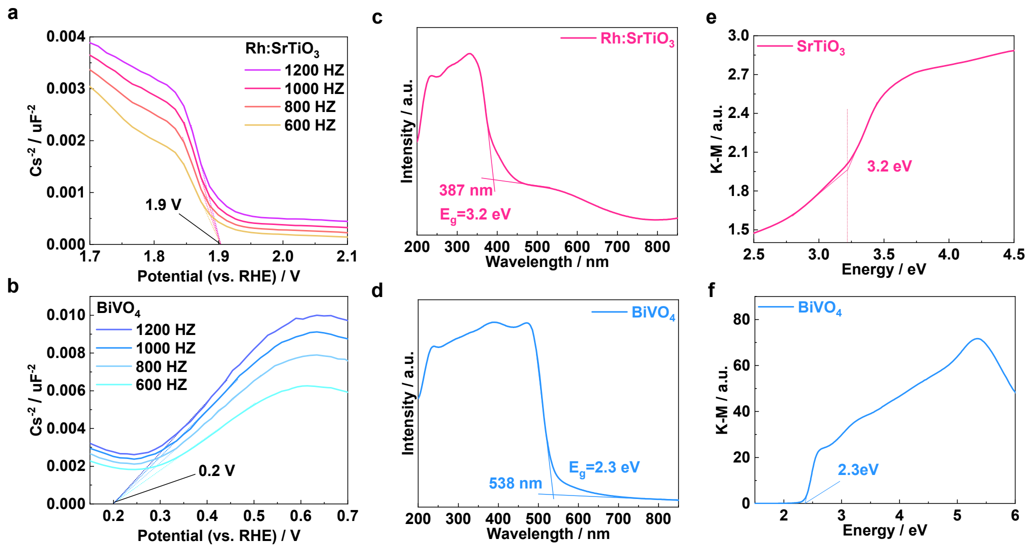

Fig. S23. The M-S curves of (**a**) Rh:$SrTiO_3$ and (**b**) faceted $BiVO_4$ samples. UV-visible absorption spectra of (**c**) Rh:$SrTiO_3$ and (**d**) faceted $BiVO_4$ samples. Tauc plots of (**e**) Rh:$SrTiO_3$ and (**f**) faceted $BiVO_4$ samples.

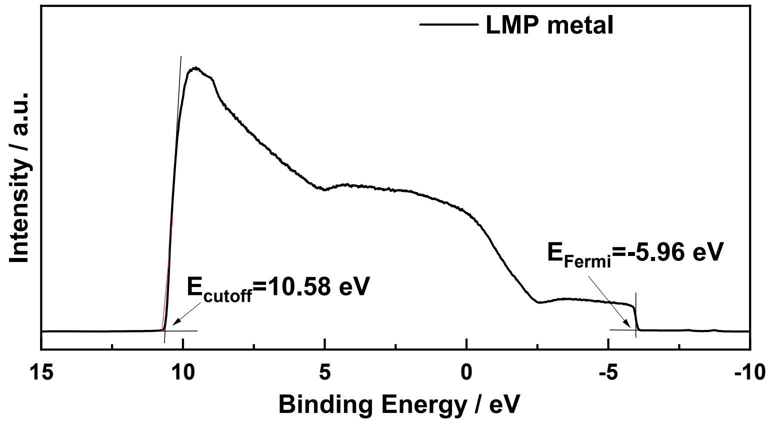

Fig. S24. The UPS spectrum of the LMP metal (Field's metal) film, showing the cutoff edge (E_{cutoff}) and Fermi edge (E_{Fermi}) of 10.58 and -5.96 eV, respectively. The work function of the LMP metal film was calculated to be 4.68 eV according to the following equation: $\Phi_{WF} = h\nu - (E_{cutoff} - E_{Fermi}) = 21.22 - (E_{cutoff} - E_F)$.

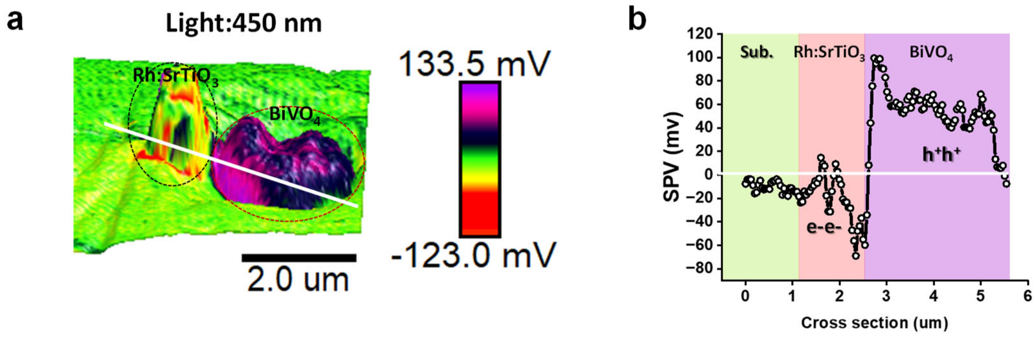

Fig. S25. (**a**) SPVM image of adjacent $BiVO_4$ and $Rh:SrTiO_3$ particles embedded in the LMP metal film. (**b**) SPV curve plotted along the line crossing over $BiVO_4$ and $Rh:SrTiO_3$ particles in (a).

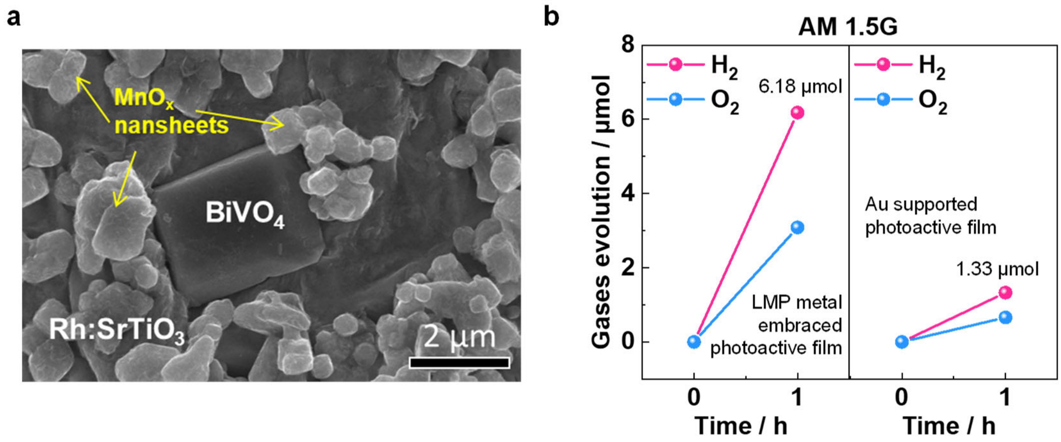

Fig. S26. (a) Photo-reduction deposition of MnO_x ($MnO_4^- + e^- \rightarrow MnO_x$) on $BiVO_4$ and $Rh:SrTiO_3$ particles embedded in the metal film. (b) Hydrogen and oxygen evolution from water splitting under AM1.5G sunlight simulator irradiation on the LMP metal embraced and Au film supported Z-scheme panel systems with area of ~10 cm^2.

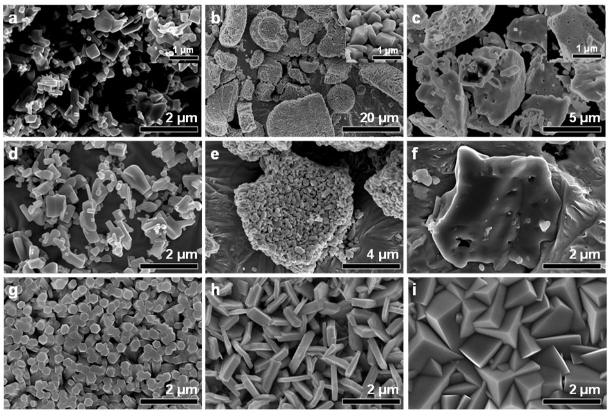

Fig. S27. SEM images of commercial (a) ZnO, (b) WO_3 and (c) Cu_2O powders, the particles-embedded photoelectrodes from commercial (d) ZnO, (e) WO_3 and (f) Cu_2O powders and the photoelectrodes of (g) ZnO nanorod arrays, (h) WO_3 nanoplate arrays and (i) Cu_2O cubic crystal arrays *in situ* grown on FTO substrates.

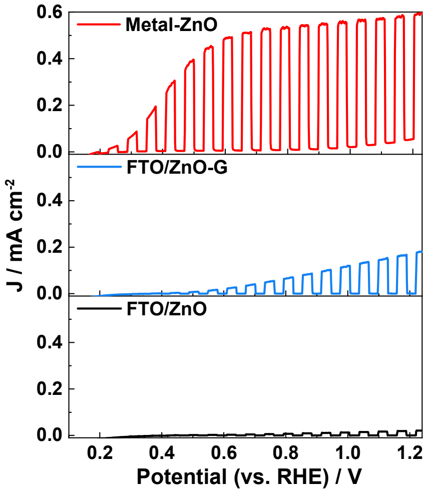

Figure S28. The polar J-V curves of the ZnO particles-embedded photoelectrode (red) from commercial powders (Metal-ZnO), ZnO photoelectrode (blue) of nanorod arrays *in situ* grown on FTO (FTO/ZnO-G) and the corresponding control ZnO photoelectrode (black) on FTO glass using traditional particle-post-assembly technique (FTO/ZnO).

Table S2 Comparison of the photoelectrochemical water splitting performance between the commercial ZnO particles-embedded photoelectrode and the representative ZnO nanostructured photoelectrodes *in situ* grown on FTO substrates.

Method	Morphology	J_{SC} at 1.23 V_{RHE} (mA cm^{-2})	Electrolyte	Ref.
Glancing-angle deposition	Nanoparticle	0.15	0.5 M $NaClO_4$ (pH 7.4)	5
Electrochemical deposition	Nanosheet arrays	0.4	0.5 M Na_2SO_4 solution	6
Vapor-transport	Branched nano-tetrapod	0.2	0.5 M Na_2SO_4 (pH 7.0)	7
Electrochemical deposition	Hierarchical nanorod arrays	0.3	0.5 M Na_2SO_4	8
Aqueous-chemical method	Nano-pencil	0.6	0.5 M Na_2SO_4	9
Hydrothermal	Caterpillar-like	0.4	0.5 M Na_2SO_4 (pH 7.0)	10
Two-step hydrothermal	Nanowire arrays	0.4	0.5 M Na_2SO_4 solution (pH 6.5)	11
Spray pyrolysis	Nanowire arrays	0.9	0.2 M Na_2SO_4 (pH 7.0)	12
Two-beam laser interference lithography	Patterned nanorod arrays	0.6	1 M Na_2SO_4 (pH 7.0)	13
Electrochemical deposition/hydrothermal	Nano-tree arrays	0.45	0.5 M Na_2SO_4	14
Hydrolysis of zinc acetate	Flower like	0.39	0.5 M Na_2SO_4	15
Anodic deposition	Nanowire arrays	0.32	0.5 M Na_2SO_4	16
DC sputtering	Nanoparticle	0.13	0.5 M Na_2SO_4	17
Electrochemical deposition	Nanosheet arrays	0.1	0.1 M Na_2SO_4	18
Two-step hydrothermal	Nanorod	0.4	0.5 M Na_2SO_4	19
Chemical vapour deposition	Nanoparticle	0.29	0.1 M Na_2SO_4 solution	20
PiP technique	Submicron particles	0.53	1 M potassium borate (pH 9)	This work

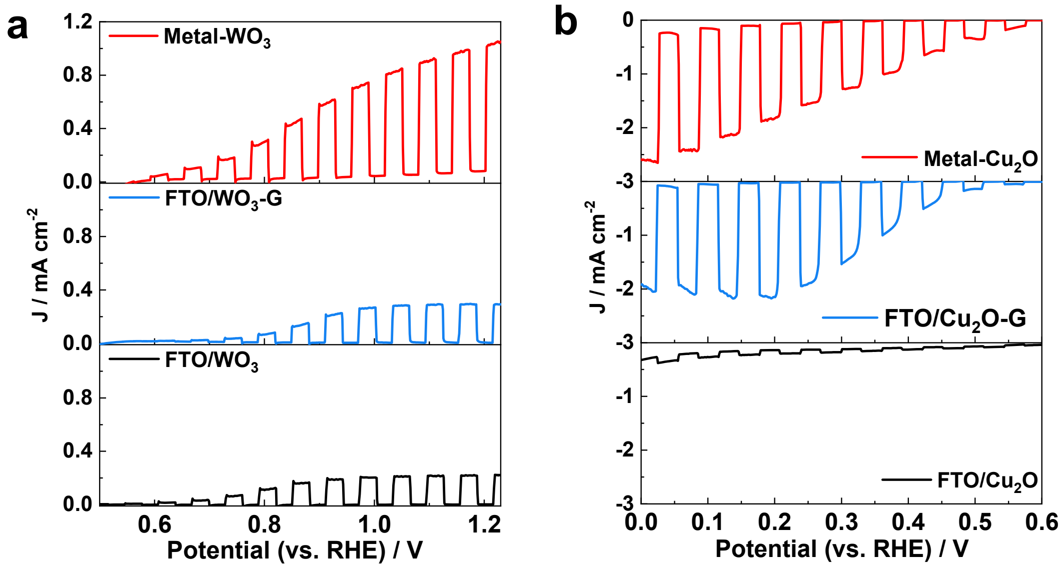

Fig. S29. (**a**) The polar J-V curves of the WO_3 particles-embedded photoelectrode (red) from commercial powders (Metal-WO_3), WO_3 photoelectrode (blue) of nanoplate arrays *in situ* grown on FTO (FTO/WO_3-G) glass substrates and the corresponding control WO_3 photoelectrode (black) on FTO glass using traditional particle-post-assembly technique (FTO/WO_3). (**b**) The polar J-V curves of the Cu_2O particles-embedded photoelectrode (red) from commercial powders (Metal-Cu_2O), Cu_2O photoelectrode (blue) of micro-cubic crystal arrays *in situ* grown on FTO (FTO/Cu_2O-G) and the corresponding control Cu_2O photoelectrode (black) on FTO using traditional particle-post-assembly technique (FTO/Cu_2O).

Table S3 Comparison of the photoelectrochemical water splitting performance between the commercial WO_3 particles-embedded photoelectrode and the representative WO_3 nanostructured photoelectrodes *in situ* grown on FTO glass substrates.

**Method**	**Morphology**	**J_{SC} at 1.23 V_{RHE} (mA cm^{-2})**	**Electrolyte**	**Ref.**
Hydrothermal	Nanoparticle	2.2	0.5 M H_2SO_4	21
Solvothermal technique	Nanoflake	1.43	0.1 M Na_2SO_4	22
Hydrothermal method	Nanorod arrays	2.26	0.5 M Na_2SO_4	23
Anodization	Nanoflake	0.9	0.5 M H_2SO_4	24
Pulsed laser deposition	Tree-like nanoporous	1.8	0.5 M phosphate buffer solution	25
Polymer-assisted direct deposition	Nanoparticle	1.45	0.1 M phosphate buffer solution	26
Pulsed laser deposition	Pyramidal-like pattern array	2.4	0.1 M H_2SO_4	27
Water bath	Nanoplate array	1.42	0.1 M Na_2SO_4	28
Hydrothermal	Nanoplate	0.43	0.1 M KPi buffer solution (pH = 7)	29
Sol-gel	Nanorod	1.2	sodium borate buffer (pH = 9)	30
Hydrothermal	Nanosheet	0.3	0.5 M Na_2SO_4	31
Anodization	Nanoporous	1.19	0.5 M Na_2SO_4 solution (pH 7.2)	32
Hydrothermal	Nanosheet	0.76	0.5 M Na_2SO_4	33
PiP technique	Microparticle	1.05	1 M potassium borate (pH 9)	This work

Table S4 Comparison of the photoelectrochemical water splitting performance between the commercial Cu_2O particles-embedded photoelectrode and the representative Cu_2O nanostructured photoelectrodes *in situ* grown on conductive substrates.

Method	Morphology	Photocurrent density	Electrolyte	Ref.
Polyol method	2-D nanosheet	-0.45 mA cm^{-2} at 0.1 V_{RHE}	0.5 M Na_2SO_4	34
Electrodeposition	(111) orientated	-2.4 mA cm^{-2} at 0.25 V_{RHE}	1 M Na_2SO_4 (pH 4.9)	35
Anodization	Nanowire	-2.3 mA cm^{-2} at 0 V_{RHE}	1 M Na_2SO_4 (pH 5)	36
Sol–gel	Small crystallite	-0.28 mA cm^{-2} at 0.05 V_{RHE}	0.1 M Na_2SO_4 (pH 5.8)	37
Wet chemical oxidation	Nanowire	-2.08 mA cm^{-2} at 0 V_{RHE}	1 M Na_2SO_4	38
Wet-chemistry method	Truncated octahedra	-4.8 mA cm^{-2} at 0 V_{RHE}	0.1 M Na_2SO_4	39
Thermal oxidation	Hierarchical porous Cu_2O	-1.5 mA cm^{-2} at 0 V_{RHE}	0.1M Na_2SO_4 (pH 7.0)	40
RF sputtering technique	Granular structure	-0.29 mA cm^{-2} at 0 V_{RHE}	0.5 M Na_2SO_4	41
Electrodeposition	Pyramid-like tripyramids	-0.58 mA cm^{-2} at 0 V_{RHE}	0.1 M Na_2SO_4	42
Electrodeposition	Pyramid	-2.9 mA cm^{-2} at 0 V_{RHE}	1 M Na_2SO_4	43
DC magnetron sputtering	rhombic	-2.7 mA cm^{-2} at 0 V_{RHE}	KPi buffer solution (pH = 5)	44
RF magnetron sputtering	Particles	-3.0 mA cm^{-2} at 0 V_{RHE}	KPi buffer solution (pH = 4.9)	45
Electrodeposition	Nanopixelated	-2.3 mA cm^{-2} at 0 V_{RHE}	KPi buffer solution (pH = 5)	46
PiP technique	Microparticle	-2.4 mA cm^{-2} at 0 V_{RHE}	1 M potassium borate (pH 9)	This work

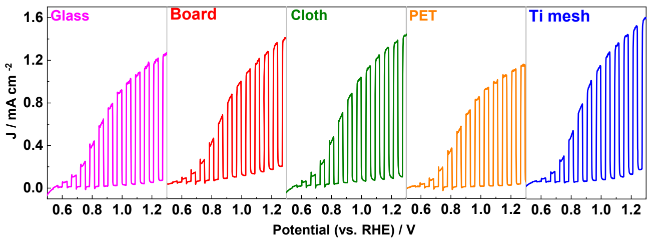

Fig. S30. The polar J-V curves of the particles-embedded photoelectrodes from commercial WO_3 particles on different substrates (e.g., glass, board, cloth, PET, and Ti mesh).

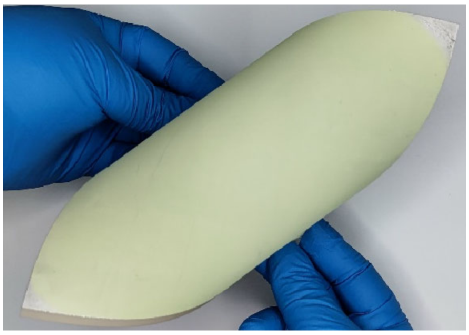

Fig. S31. Optical image of the photoelectrode film of LMP metal embraced WO_3 particles assembled on a large-size (100 mm × 100 mm) PET substrate under bending.

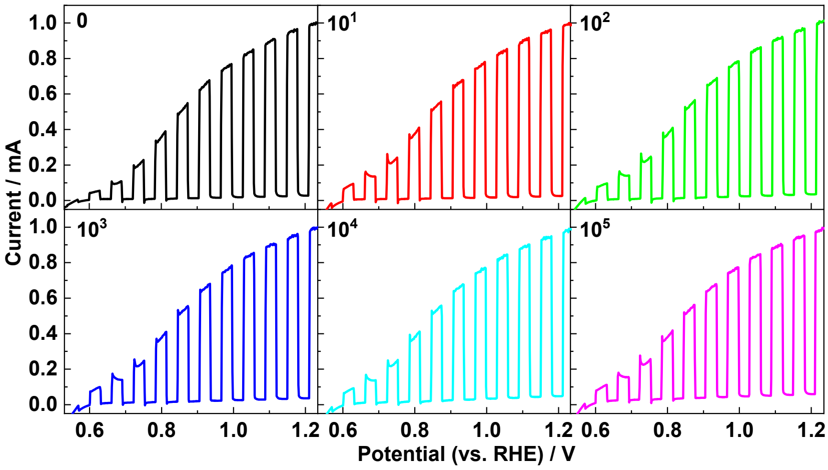

Fig. S32. The polar I-V curves of the WO_3 particle-embedded photoelectrode assembled on the flexible PET substrate recorded after every magnitude bending cycles.

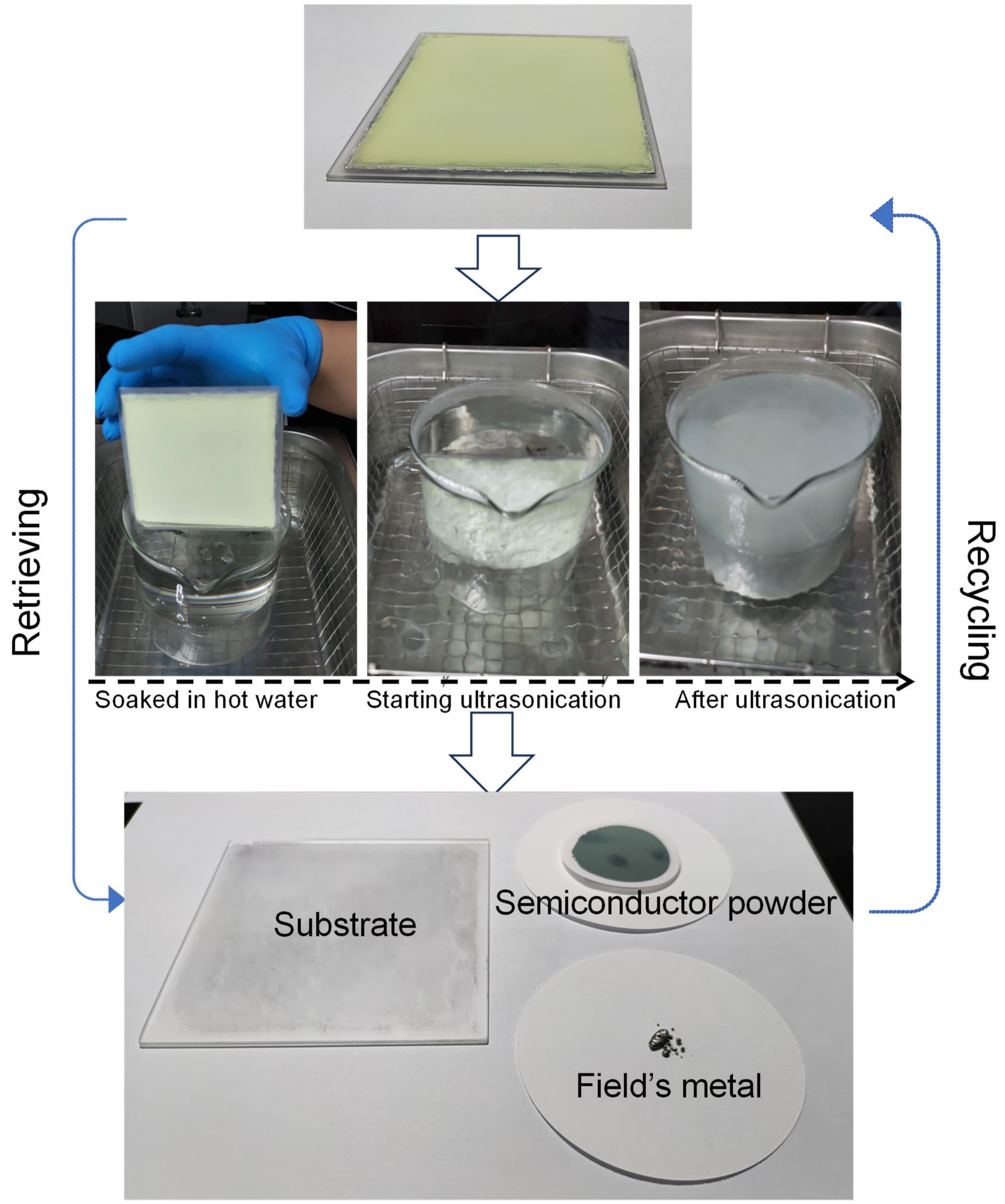

Fig. S33. The optical images of the large size (10×10 cm) particles-embedded photoelectrode from commercial WO_3 particles and the retrieved raw materials by ultrasonicating it in a hot water bath.

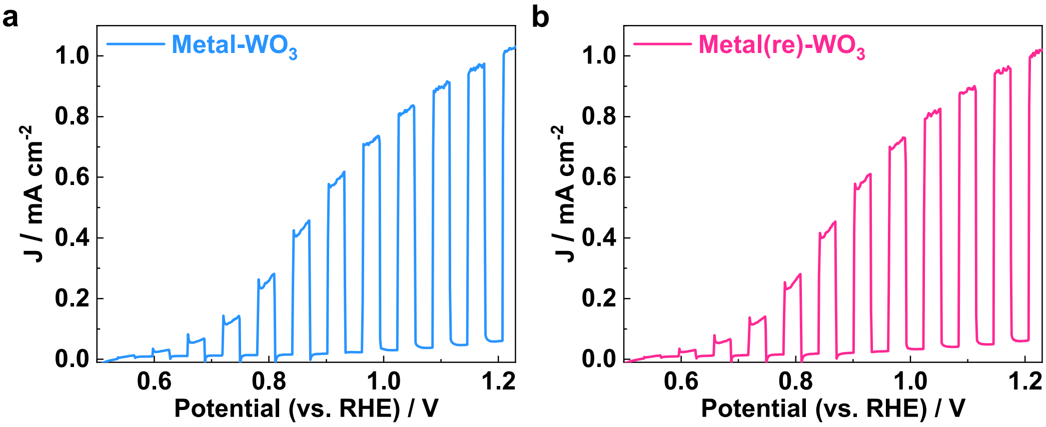

Fig. S34. The polar J-V curves recorded from the WO_3 particles embedded photoelectrodes assembled using (a) fresh and (b) retrieved metals.

References

1 Kim, C. W., Son, Y. S., Kang, M. J., Kim, D. Y. & Kang, Y. S. (040)-crystal facet engineering of $BiVO_4$ plate photoanodes for solar fuel production. *Adv. Energy Mater.* **6**, 1501754 (2016). https://doi.org/10.1002/aenm.201501754

2 Wang, S., Chen, P., Yun, J.-H., Hu, Y. & Wang, L. An electrochemically treated $BiVO_4$ photoanode for efficient photoelectrochemical water splitting. *Angew. Chem. Int. Ed.* **56**, 8500-8504 (2017). https://doi.org/10.1002/anie.201703491

3 Gao, B. *et al.* Selective deposition of Ag_3PO_4 on specific facet of $BiVO_4$ nanoplate for enhanced photoelectrochemical performance. *Sol. RRL* **2**, 1800102 (2018). https://doi.org/10.1002/solr.201800102

4 Li, D. *et al.* Crystallographic-orientation-dependent charge separation of $BiVO_4$ for solar water oxidation. *ACS Energy Lett.* **4**, 825-831 (2019). https://doi.org/10.1021/acsenergylett.9b00153

5 Wolcott, A., Smith, W. A., Kuykendall, T. R., Zhao, Y. & Zhang, J. Z. Photoelectrochemical study of nanostructured ZnO thin films for hydrogen generation from water splitting. *Adv. Funct. Mater.* **19**, 1849-1856 (2009). https://doi.org/10.1002/adfm.200801363

6 Hsu, Y.-K., Lin, Y.-G. & Chen, Y.-C. Polarity-dependent photoelectrochemical activity in ZnO nanostructures for solar water splitting. *Electrochem. Commun.* **13**, 1383-1386 (2011). https://doi.org/10.1016/j.elecom.2011.08.016

7 Qiu, Y., Yan, K., Deng, H. & Yang, S. Secondary branching and nitrogen doping of ZnO nanotetrapods: building a highly active network for photoelectrochemical water splitting. *Nano Lett.* **12**, 407-413 (2012). https://doi.org/10.1021/nl2037326

8 Yao, C. *et al.* Enhanced photoelectrochemical performance of hydrogenated ZnO hierarchical nanorod arrays. *J. Power Sources* **237**, 295-299 (2013). https://doi.org/10.1016/j.jpowsour.2013.02.062

9 Lv, R. *et al.* Facile synthesis of ZnO nanopencil arrays for photoelectrochemical water splitting. *Nano Energy* **7**, 143-150 (2014). https://doi.org/10.1016/j.nanoen.2014.04.020

10 Li, Q., Sun, X., Lozano, K. & Mao, Y. Facile and scalable synthesis of “caterpillar-like” ZnO nanostructures with enhanced photoelectrochemical water-splitting effect. *J. Phys. Chem. C* **118**, 13467-13475 (2014). https://doi.org/10.1021/jp503155c

11 Chen, H. *et al.* Epitaxial growth of ZnO nanodisks with large exposed polar facets on nanowire arrays for promoting photoelectrochemical water splitting. *Small* **10**, 4760-4769 (2014). https://doi.org/10.1002/smll.201401298

12 Jiang, C., Moniz, S. J. A., Khraisheh, M. & Tang, J. Earth-abundant oxygen evolution catalysts coupled onto ZnO nanowire arrays for efficient photoelectrochemical water cleavage. *Chem. Eur. J.* **20**, 12954-12961 (2014). https://doi.org/10.1002/chem.201403067

13 Hu, Y. *et al.* Large-scale patterned ZnO nanorod arrays for efficient photoelectrochemical water splitting. *Appl. Surf. Sci.* **339**, 122-127 (2015). https://doi.org/10.1016/j.apsusc.2015.02.074

14 Ren, X. *et al.* Photoelectrochemical water splitting strongly enhanced in fast-grown ZnO nanotree and nanocluster structures. *J. Mater. Chem. A* **4**, 10203-10211 (2016). https://doi.org/10.1039/C6TA02788A

15 Sohila, S., Rajendran, R., Yaakob, Z., Teridi, M. A. M. & Sopian, K. Photoelectrochemical water splitting performance of flower like ZnO nanostructures synthesized by a novel chemical method. *J. Mater. Sci.: Mater. Electron.* **27**, 2846-2851 (2016). https://doi.org/10.1007/s10854-015-4100-2

16 Huang, M.-C., Wang, T., Wu, B.-J., Lin, J.-C. & Wu, C.-C. Anodized ZnO nanostructures for photoelectrochemical water splitting. *Appl. Surf. Sci.* **360**, 442-450 (2016). https://doi.org/10.1016/j.apsusc.2015.09.174

17 Bai, S. *et al.* NiFePB-modified ZnO/$BiVO_4$ photoanode for PEC water oxidation. *Dalton Trans.* **52**, 5760-5770 (2023). https://doi.org/10.1039/D3DT00013C

18 Khan, H. R. *et al.* Superior photoelectrochemical performance by antimony-doped ZnO thin films by AACVD approach. *Bull. Mater. Sci.* **45**, 55 (2022). https://doi.org/10.1007/s12034-021-02624-x

19 Salih, A. K., Phillips, M. R. & Ton-That, C. Enhanced solar-driven water splitting performance using oxygen vacancy rich ZnO photoanodes. *Sol. Energy Mater. Sol. Cells* **259**, 112436 (2023). https://doi.org/10.1016/j.solmat.2023.112436

20 Wang, D. *et al.* Piezoelectric polarization induced by dual piezoelectric materials ZnO nanosheets/MoS_2 heterostructure for enhancing photoelectrochemical water splitting. *J. Colloid Interface Sci.* **653**, 1166-1176 (2024). https://doi.org/10.1016/j.jcis.2023.09.157

21 Li, W., Li, J., Wang, X., Ma, J. & Chen, Q. Photoelectrochemical and physical properties of WO_3 films obtained by the polymeric precursor method. *Int. J. Hydrog. Energy* **35**, 13137-13145 (2010). https://doi.org/10.1016/j.ijhydene.2010.09.011

22 Su, J., Feng, X., Sloppy, J. D., Guo, L. & Grimes, C. A. Vertically aligned WO_3 nanowire arrays grown directly on transparent conducting oxide coated glass: synthesis and photoelectrochemical properties. *Nano Lett.* **11**, 203-208 (2011). https://doi.org/10.1021/nl1034573

23 Kalanur, S. S., Hwang, Y. J., Chae, S. Y. & Joo, O. S. Facile growth of aligned WO_3 nanorods on FTO substrate for enhanced photoanodic water oxidation activity. *J. Mater. Chem. A* **1**, 3479-3488 (2013). https://doi.org/10.1039/C3TA01175E

24 Qi, H. *et al.* Triple-layered nanostructured WO_3 photoanodes with enhanced photocurrent generation and superior stability for photoelectrochemical solar energy conversion. *Nanoscale* **6**, 13457-13462 (2014). https://doi.org/10.1039/C4NR03982C

25 Shin, S. *et al.* A tree-like nanoporous WO_3 photoanode with enhanced charge transport efficiency for photoelectrochemical water oxidation. *J. Mater. Chem. A* **3**, 12920-12926 (2015). https://doi.org/10.1039/C5TA00823A

26 Kim, J. H., Lee, B. J., Wang, P., Son, M. H. & Lee, J. S. Facile surfactant driven fabrication of transparent WO_3 photoanodes for improved photoelectrochemical properties. *Appl. Catal. A* **521**, 233-239 (2016). https://doi.org/10.1016/j.apcata.2016.01.003

27 Fàbrega, C. *et al.* Efficient WO_3 photoanodes fabricated by pulsed laser deposition for photoelectrochemical water splitting with high faradaic efficiency. *Appl. Catal. B* **189**, 133-140 (2016). https://doi.org/10.1016/j.apcatb.2016.02.047

28 Zeng, Q. *et al.* Preparation of vertically aligned WO_3 nanoplate array films based on peroxotungstate reduction reaction and their excellent photoelectrocatalytic performance. *Appl. Catal. B* **202**, 388-396 (2017). https://doi.org/10.1016/j.apcatb.2016.09.045

29 Li, Y. *et al.* Fluorine-doped iron oxyhydroxide cocatalyst: promotion on the WO_3 photoanode conducted photoelectrochemical water splitting. *Appl. Catal. B* **304**, 120995 (2022). https://doi.org/10.1016/j.apcatb.2021.120995

30 Fang, W., Lin, Y., Xv, R. & Fu, L. Boosting photoelectrochemical performance of $BiVO_4$ photoanode by synergistic effect of $WO_3/BiVO_4$ heterojunction construction and NiOOH water oxidation cocatalyst modification. *ACS Appl. Energy Mater.* **5**, 11402-11412 (2022). https://doi.org/10.1021/acsaem.2c01869

31 Li, H. *et al.* Oxygen vacancy-mediated WO_3 phase junction to steering photogenerated charge separation for enhanced water splitting. *J. Adv. Ceram.* **11**, 1873-1888 (2022). https://doi.org/10.1007/s40145-022-0653-8

32 Xia, M. *et al.* High-voltage etching-induced terrace-like WO_3 photoanode for efficient photoelectrochemical water splitting. *ACS Appl. Energy Mater.* **6**, 8717-8728 (2023). https://doi.org/10.1021/acsaem.3c01164

33 Khan, H. *et al.* Sustained water oxidation with surface- and interface-engineered $WO_3/BiVO_4$ heterojunction photoanodes. *ACS Appl. Energy Mater.* **5**, 15788-15798 (2022). https://doi.org/10.1021/acsaem.2c03345

34 Ma, L. *et al.* Aligned 2-D nanosheet Cu_2O film: oriented deposition on Cu foil and its photoelectrochemical property. *J. Phys. Chem. C* **112**, 18916-18922 (2008). https://doi.org/10.1021/jp807219u

35 Paracchino, A., Laporte, V., Sivula, K., Grätzel, M. & Thimsen, E. Highly active oxide photocathode for photoelectrochemical water reduction. *Nat. Mater.* **10**, 456-461 (2011). https://doi.org/10.1038/nmat3017

36 Dubale, A. A. *et al.* The synergetic effect of graphene on Cu_2O nanowire arrays as a highly efficient hydrogen evolution photocathode in water splitting. *J. Mater. Chem. A* **2**, 18383-18397 (2014). https://doi.org/10.1039/C4TA03464C

37 Lim, Y.-F., Chua, C. S., Lee, C. J. J. & Chi, D. Sol–gel deposited Cu_2O and CuO thin films for photocatalytic water splitting. *Phys. Chem. Chem. Phys.* **16**, 25928-25934 (2014). https://doi.org/10.1039/C4CP03241A

38 Bai, J. *et al.* A novel 3D ZnO/Cu_2O nanowire photocathode material with highly efficient photoelectrocatalytic performance. *J. Mater. Chem. A* **3**, 22996-23002 (2015). https://doi.org/10.1039/C5TA07583A

39 Jin, Z., Hu, Z., Yu, J. C. & Wang, J. Room temperature synthesis of a highly active Cu/Cu_2O photocathode for photoelectrochemical water splitting. *J. Mater. Chem. A* **4**, 13736-13741 (2016). https://doi.org/10.1039/C6TA05274F

40 Ma, X., Zhang, J., Wang, B., Li, Q. & Chu, S. Hierarchical Cu_2O foam/g-C_3N_4 photocathode for photoelectrochemical hydrogen production. *Appl. Surf. Sci.* **427**, 907-916 (2018). https://doi.org/10.1016/j.apsusc.2017.09.075

41 Tawfik, W. Z., Hassan, M. A., Johar, M. A., Ryu, S.-W. & Lee, J. K. Highly conversion efficiency of solar water splitting over p-Cu_2O/ZnO photocatalyst grown on a metallic substrate. *J. Catal.* **374**, 276-283 (2019). https://doi.org/10.1016/j.jcat.2019.04.045

42 Wang, P. *et al.* Decorating Cu_2O photocathode with Cu/Al bimetallic layer for enhanced photoelectrochemical water splitting. *Int. J. Energy Res.* **46**, 16991-17002 (2022). https://doi.org/10.1002/er.8363

43 Chang, T.-K., Huang, Y.-S., Chen, H.-Y. & Liao, C.-N. Photoelectrochemical enhancement of Cu_2O by a Cu_2Te hole transmission interlayer. *ACS Appl. Mater. Interfaces* **14**, 48540-48546 (2022). https://doi.org/10.1021/acsami.2c10448

44 Qin, C. *et al.* Fabricating high-quality Cu_2O photocathode by magnetron sputtering: insight into defect states and charge carrier collection in Cu_2O. *ACS Appl. Energy Mater.* **5**, 14410-14422 (2022). https://doi.org/10.1021/acsaem.2c02974

45 Kalanur, S. S., Lee, Y. J. & Seo, H. Enhanced and stable photoelectrochemical H_2 production using a engineered nano multijunction with Cu_2O photocathode. *Mater. Today Chem.* **26**, 101031 (2022). https://doi.org/10.1016/j.mtchem.2022.101031

46 Lee, J. & Oh, J. Nanopixelated cuprous oxide photocathodes for durable photoelectrochemical water splitting. *ACS Energy Lett.* **7**, 3244-3250 (2022). https://doi.org/10.1021/acsenergylett.2c01540
